# Social status, facial structure, and assertiveness in brown capuchin monkeys

**DOI:** 10.3389/fpsyg.2014.00567

**Published:** 2014-06-11

**Authors:** Justin M. Carré

**Affiliations:** Department of Psychology, Nipissing UniversityNorth Bay ON, Canada

**Keywords:** facial width-to-height ratio, dominance, aggression, social status, testosterone

A recent paper by Lefevre et al. (2014) in PLoS One reported that individual differences in facial structure predicted assertiveness in brown capuchin monkeys (*Sapajus spp*). Specifically, variation in the facial width-to-height ratio (fWHR) was positively correlated with alpha status and a composite measure of assertiveness. This novel finding adds to a growing body of evidence indicating that variation in facial structure reliably maps onto individual differences in dominance-related phenotypes.

Research into fWHR was propelled by an anthropological study of human skulls indicating that fWHR was a size-independent sexually dimorphic feature of the human skull that arose around puberty coincident with the rise in pubertal testosterone (Weston et al., 2007). To the extent that variation in the fWHR is under the influence of pubertal testosterone, and that pubertal testosterone organizes the neural circuitry underlying sexually dimorphic behaviors, we tested the hypothesis that this metric would map onto human aggression. Initial support for this hypothesis came from a series of studies in which fWHR was positively correlated with aggressive behavior in men tested in a laboratory task as well as in varsity and professional hockey players (Carré and McCormick, 2008). Since this publication, several studies have found that this metric maps onto other conceptually similar phenotypes. For instance, fWHR is associated with unethical behavior (Haselhuhn and Wong, 2012; Geniole et al., 2014), non-reciprocation of trust (Stirrat and Perrett, 2010), psychopathic traits (Geniole et al., 2014), fighting abilities (Stirrat et al., 2012; Zilioli et al., accepted), explicit prejudice (Hehman et al., 2013) and selfishness (Haselhuhn et al., 2013).

Despite these findings, there have been some non-replications. Ozener (2012) first reported that fWHR was not sexually dimorphic and did not predict aggression as assessed using a self-report measure. The lack of a sex difference in fWHR has now been reported in several relatively large-scale studies (e.g., Lefevre et al., 2012). In addition, a study with a larger sample of professional hockey players reported that fWHR was only marginally positively correlated (*p* = 0.057) with aggression (Deaner et al., 2012). Finally, in a Mexican sample, fWHR did not differ between males convicted of violent vs. non-violent crimes (Gómez-Valdés et al., 2013). What may account for such discrepant findings? Were the original findings Type I errors? I believe this is an unlikely explanation given that several independent laboratories have found associations between fWHR and traits that are conceptually linked to dominance and aggression (see above). Another possibility is that the link between fWHR and dominance behavior is moderated by social context. Consistent with this idea, we recently reported that the relationship between fWHR and aggressive behavior in men was moderated by subjective and objective measures of social status (Goetz et al., 2013). Here, fWHR was positively correlated with aggression, but only among relatively low status men (Goetz et al., 2013).

In their paper, Lefevre et al. (2014) reported positive correlations between fWHR, alpha status, and “assertiveness.” The latter construct consisted of traits such as bullying, aggression, dominance, jealousy, and stinginess. This is a novel finding, documenting for the first time a link between fWHR and complex social behavior in a non-human primate. Although the authors reported that alpha status did not significantly moderate the relationship between fWHR and assertiveness (*p* = 0.09), a careful examination of Figure 4 from Lefevre et al. (2014) certainly suggested that the effects were driven by non-alpha (i.e., low-ranking) monkeys. Indeed, bivariate correlations performed separately for alpha and non-alpha monkeys indicated that the relationship between fWHR and assertiveness was significant in non-alpha [*r*_(23)_ = 0.54, *p* = 0.005], but not alpha monkeys [*r*_(16)_ = 0.02, *p* = 0.94]. I decided to perform a re-analysis of Lefevre and colleagues' data which were freely available on the PLoS One website (http://www.plosone.org/article/info%3Adoi%2F10.1371%2Fjournal.pone.0093369) to investigate the extent to which the link between fWHR and assertiveness was driven by low status monkeys. In this model, assertiveness was the dependent variable and I included fWHR and alpha status on Step 1 and the fWHR-×-alpha status interaction on Step 2. As per Lefevre et al. (2014), I also included sex and age as covariates in the model, although they emerged as non-significant predictors of assertiveness (*p* = 0.42 and *p* = 0.13, respectively).

Results revealed main effects of alpha status (*B* = −1.32, *SE* = 0.30, *p* < 0.01) and fWHR (*B* = 3.02, *SE* = 0.89, *p* < 0.03) and a trend for a fWHR-×-alpha status interaction (*B* = 2.89, *SE* = 1.70, *p* = 0.096). Lefevre et al. (2014) did not probe this interaction, presumably because it did not reach the conventional level of statistical significance (i.e., *p* < 0.05). However, the lack of statistical significance is almost certainly due to a lack of statistical power given the small sample size (*n* = 43). Because previous work has found that the relationship between fWHR and aggressive behavior is specific to individuals with relatively low social status (Goetz et al., 2013), I further probed the marginal interaction with simple slopes analyses. Consistent with previous work in humans, these analyses indicated that fWHR was positively correlated with assertiveness in non-alpha monkeys (*B* = 3.24, *SE* = 1.22, *p* < 0.02) but not alpha monkeys (*B* = 0.35, *SE* = 1.23, *p* = 0.78) (see Figure 1). Notably, there were no sex-×-alpha status (*p* = 0.65), sex-×-fWHR (*p* = 0.84), or sex-×-alpha status-×-fWHR interactions (*p* = 0.15), suggesting that the relationships between fWHR, alpha status, and assertiveness were not moderated by sex.

**Figure 1:**
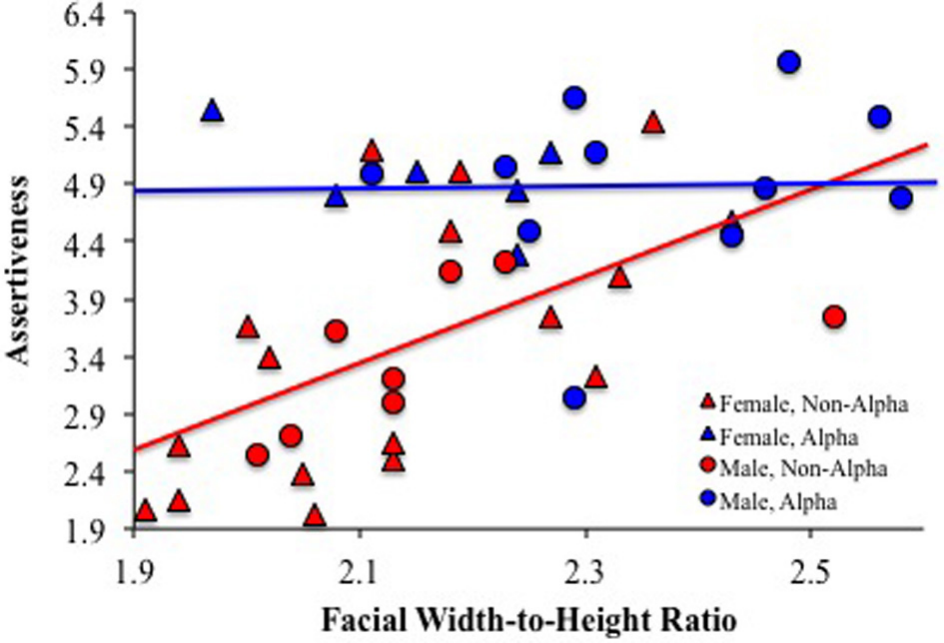
**Alpha status moderates the relationship between facial width-to-height ratio and assertiveness in capuchin monkeys**. Data from Lefevre et al. (2014). Note: the blue regression line represents the relationship between facial width-to-height ratio and assertiveness in alpha monkeys and the red regression line represents the relationship between facial width-to-height ratio and assertiveness in non-alpha monkeys. Alpha males (*n* = 11), *r* = 0.22, *p* = 0.52; Non-alpha males (*n* = 8), *r* = 0.58, *p* = 0.13; Alpha females (*n* = 7), *r* = −0.62, *p* = 0.14; Non-alpha females (*n* = 17), *r* = 0.58, *p* = 0.01.

This re-analysis of Lefevre et al.'s (2014 data suggests that one's current social circumstance has an important effect on whether variation in fWHR predicts assertive behavior. Under favorable social circumstances (i.e., high social rank), fWHR is irrelevant to assertiveness. In contrast, under poor social circumstances, fWHR is positively correlated with assertiveness. Here, low-ranking monkeys are relatively submissive (or unassertive), but only to the extent that they have low fWHRs. In contrast, low-ranking monkeys with large fWHRs are just as assertive as high-ranking monkeys. What may explain such findings? To the extent that fWHR is a positive correlate of fighting abilities in monkeys, as it is in humans (e.g., Stirrat et al., 2012; Zilioli et al., accepted), low-ranking monkeys with large fWHRs may be more likely to successfully implement assertive behaviors in their social interactions, and thus, the net benefits of engaging in such behaviors (e.g., increased access to food, mating opportunities) may outweigh the costs. In contrast, low-ranking monkeys with relatively small fWHRs, who are presumably weaker, may be less likely to successfully implement assertive behavioral strategies. This possibility is speculative, and the extent to which fWHR is associated with physical strength/fighting abilities in brown capuchin monkeys will require further investigation.

One important finding from Lefevre et al. (2014) and the current re-analysis was that the effects observed were independent of sex. This contrasts human work indicating that associations between fWHR, social status, and aggression are only found in men (Goetz et al., 2013). As suggested by Lefevre et al. (2014), the sex independent effects of fWHR may be due to the fact that in brown capuchin monkeys, females commonly engage in aggression with other males and females, suggesting that dominance behavior may be less sexually differentiated in this species then in other primate species. Also, the composite measure of assertiveness used by Lefevre et al. (2014) tapped into constructs related to aggression, bullying, stinginess, dominance, jealousy and irritability. Thus, it remains unclear which factor(s) most closely mapped onto fWHR and alpha status, and whether such links were specific to males or females. One important limitation of this work is the small number of capuchin monkeys used, which renders significant moderation effects difficult to detect. Although my re-analysis of Lefevre et al. (2014) strongly suggests that the relationship between fWHR and assertiveness is primarily driven by non-alpha monkeys, these findings should be interpreted with some caution given that the alpha status × fWHR interaction did not reach the conventional level of significance (*p* = 0.096). Thus, future work will require a larger sample to verify the extent to which fWHR maps onto dominance-related traits, whether such effects are moderated by social status, and whether relationships between fWHR, social status, and assertiveness hold across males and females.

In summary, these findings in brown capuchin monkeys, along with work in humans (Goetz et al., 2013) highlight the importance of considering social status as a moderator of the relationship between fWHR and dominance related behaviors.

## Conflict of interest statement

The author declares that the research was conducted in the absence of any commercial or financial relationships that could be construed as a potential conflict of interest.
